# SOCS2-Induced Proteasome-Dependent TRAF6 Degradation: A Common Anti-Inflammatory Pathway for Control of Innate Immune Responses

**DOI:** 10.1371/journal.pone.0038384

**Published:** 2012-06-05

**Authors:** Cortez McBerry, Rosa Maria Salazar Gonzalez, Nathaniel Shryock, Alexandra Dias, Julio Aliberti

**Affiliations:** Divisions of Molecular Immunology and Pulmonary Medicine, Cincinnati Children’s Hospital Medical Center, Cincinnati, Ohio, United States of America; National Council of Sciences (CONICET), Argentina

## Abstract

Pattern recognition receptors and receptors for pro-inflammatory cytokines provide critical signals to drive the development of protective immunity to infection. Therefore, counter-regulatory pathways are required to ensure that overwhelming inflammation harm host tissues. Previously, we showed that lipoxins modulate immune response during infection, restraining inflammation during infectious diseases in an Aryl hydrocarbon receptor (AhR)/suppressors of cytokine signaling (SOCS)2-dependent-manner. Recently, Indoleamine-pyrrole 2,3- dioxygenase (IDO)-derived tryptophan metabolites, including L-kynurenine, were also shown to be involved in several counter-regulatory mechanisms. Herein, we addressed whether the intracellular molecular events induced by lipoxins mediating control of innate immune signaling are part of a common regulatory pathway also shared by L-kynurenine exposure. We demonstrate that Tumor necrosis factor receptor-associated factor (TRAF)6 – member of a family of adapter molecules that couple the TNF receptor and interleukin-1 receptor/Toll-like receptor families to intracellular signaling events essential for the development of immune responses – is targeted by both lipoxins and L-kynurenine via an AhR/SOCS2-dependent pathway. Furthermore, we show that LXA_4_- and L-kynurenine-induced AhR activation, its subsequent nuclear translocation, leading SOCS2 expression and TRAF6 Lys47-linked poly-ubiquitination and proteosome-mediated degradation of the adapter proteins. The in vitro consequences of such molecular interactions included inhibition of TLR- and cytokine receptor-driven signal transduction and cytokine production. Subsequently, in vivo proteosome inhibition led to unresponsiveness to lipoxins, as well as to uncontrolled pro-inflammatory reactions and elevated mortality during toxoplasmosis. In summary, our results establish proteasome degradation of TRAF6 as a key molecular target for the anti-inflammatory pathway triggered by lipoxins and L-kynurenine, critical counter-regulatory mediators in the innate and adaptive immune systems.

## Introduction

Lipoxins (LX) are trihydroxytetraene-containing arachidonic acid mediators that down-modulate and promote the resolution of inflammatory processes [1]–[7]. Lipoxins play a relevant counter-regulatory role in a growing list of mouse models of infectious diseases, including *Mycobacterium tuberculosis* and *Toxoplasma gondii* [2], [3], [7], [8]. Similarly, deficient LX-mediated counter-regulation has been linked to the pathogenesis of inflammatory diseases such as severe asthma [9], cystic fibrosis lung disease [3] and periodontal disease [7]. Lipoxin A_4_ (LXA_4_) is the most prominent mediator of this class. Although it is still not clear how these mediators operate at the intracellular level, LX analog administration has showed beneficial effects in several rodent models of inflammatory pathology suggesting a therapeutic promise for specific harnessing of the biological activities of these potent lipid mediators. Their molecular mechanism(s) of action remain under-defined, however.

Tryptophan catabolism into kynurenine by IDO functions as a counter-regulatory pathway mediating potent suppression of T cell responses *in vitro* and *in vivo* [10], however the molecular mechanisms remain to be fully defined. Inhibition of T cell responses by IDO-expressing dendritic cells is thought to play an important physiological role in suppressing the development and expression of autoimmune and allergic diseases [10]. Moreover, IDO is expressed by many tumors, as well as by a subpopulation of dendritic cells in tumor-draining lymph nodes. Consequently, IDO inhibition can rescue anergic, tumor antigen-specific T cell effector function, inhibiting tumor growth in mouse models [11], [12]. Sustained IDO activation is also thought to be an important cause of immunosuppression in HIV infection [13]. During toxoplasmosis, it has been shown that IDO activation is critical for controlling intracellular pathogen multiplication, probably via tryptophan starvation [14]. IDO expression can be regulated in diverse cell types by pathogen- and host-derived inflammatory signals, including pro-inflammatory cytokines (i.e. IFN-γ), Toll-like receptor ligands (e.g., lipopolysaccharide), and interactions between immune cells (e.g., the engagement of costimulatory molecules on antigen presenting cells by CTLA-4) [10]. The potential for therapeutic exploitation of physiological IDO activity (in autoimmune disease and transplantation) and therapeutic targeting of pathophysiological IDO activity (in cancer and HIV) is currently under active investigation.

IDO-derived tryptophan metabolites were previously shown to interact with the nuclear receptor, aryl hydrocarbon receptor (AhR) [15]–[17]. AhR is present in the cytoplasm associated with chaperones, such as HSP90. Upon ligand binding, AhR is released from HSP90 and pairs with the nuclear transporter – ARNT – translocates to the nucleus and activates gene transcription of dioxin-responsive elements (DRE) sequences within promoter regions of several genes, including cytochrome p450 gene family members, genes involved in control of cell cycle and cell death, including SOCS2. We recently showed that SOCS2 is an important AhR-dependent intracellular mediator of the anti-inflammatory actions of lipoxins during in vivo *T. gondii* infection [18]. Although there is previous evidence for lipoxin triggering of AhR [19], such results have not been further confirmed to date. SOCS proteins are known to inhibit receptor-mediated signal transduction via several pathways, including inhibition of tyrosine phosphorylation by allosteric blockage (e.g., of JAK proteins) and induction of proteasomal degradation by promoting polyubiquitinylation (e.g., of STAT proteins) [20].

Taken together there is strong evidence that both lipoxins and the tryptophan metabolite L-kynurenine share a common intracellular pathway via induction of SOCS2. The present study was aimed to define the molecular target(s) and mechanism(s) of action of lipoxin- and L-kynurenine-induced SOCS2. The results presented here demonstrate that lipoxins and L-kynurenine induce SOCS2-dependent ubiquitinylation and proteasomal degradation of TRAF6, hindering pro inflammatory cytokine expression by dendritic cells. Further, these results indicate that the proteosome-dependent pathway is triggered by lipoxins to modulate innate immune signaling and is critical to restrain the in vivo inflammatory responses during infection with *T. gondii*.

## Results

### LXA_4_ and L-kynurenine Inhibit Pro-inflammatory Cytokine Responses in vitro

**Figure 1:**
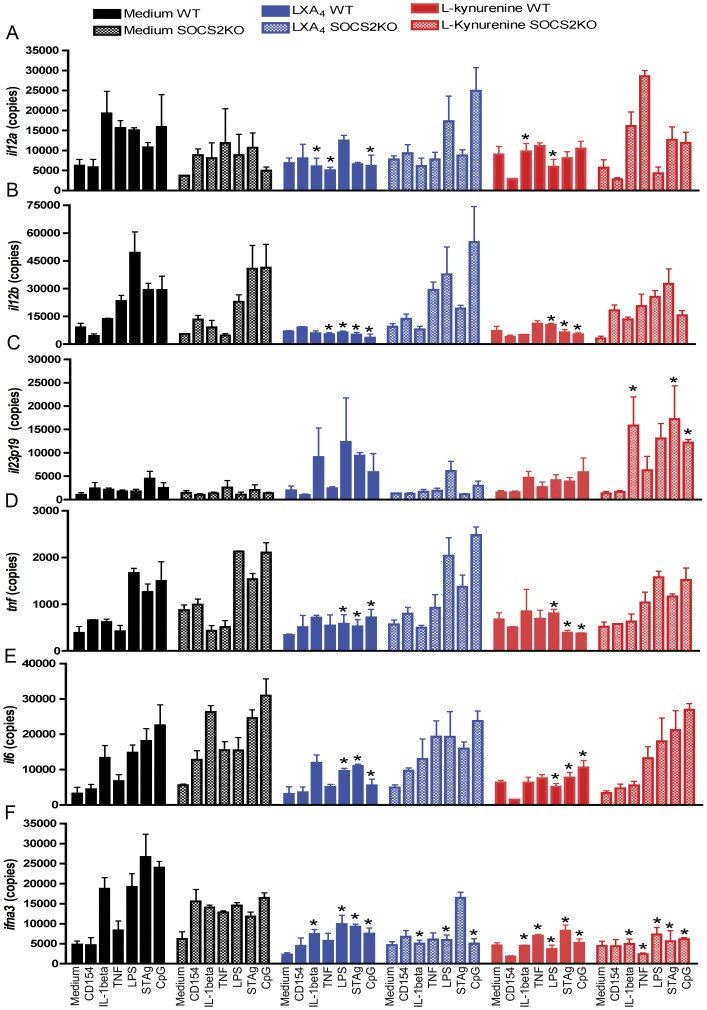
LXA_4_ and L-kynurenine suppress pro-inflammatory gene induction in a SOCS2-dependent manner. WT (filled symbols) or SOCS2-KO (empty symbols) spleens were harvested and CD11c+ cells purified. Cells were incubated in the presence of medium alone (black squares) or with LXA_4_ (3 nM – blue triangles) and L-kynurenine (60 µM – red circles) for 6 hrs, followed by stimulation with CD154, IL-1β, TNF, LPS, STAg or CpG-oligos (1 µg/mL) for 4 hrs. Cells were lized, total RNA obtained and real-time RT-PCR reaction performed for the following genes: *il12a* (A), *il12b* (B), *il23p19* (C), *tnf* (D), *il6* (E) and *ifna3* (F). Data shown represents normalized values against β-actin expression levels and are representative of three independent assays performed. Asterisks indicate statistically significant differences between the medium versus LXA_4_ or L-kynurenine treated samples (*p*<0.05).

Considering our previous finding that LXA_4_ exposure triggers AhR-dependent SOCS2 expression that tryptophan metabolites, including L-kynurenine trigger AhR, we raised the question whether both LXA_4_ and L-kynurenine could initiate a similar anti-inflammatory profile in dendritic cells. Therefore we tested if pre-exposure to LXA_4_ (blue bars) or L-kynurenine (red bars) could inhibit in a SOCS2-dependent manner, the pro-inflammatory response after incubation with a panel of microbial/endogenous ligands (microbial products, CD154, IL-1β). Our results show (Figures 1A through E) that both mediators tested caused a broad inhibitory action effectively blocking several inter-related pro-inflammatory pathways, including TLR/MyD88, TLR/TRIF, IL-1R/MyD88, CD40/CD154. Moreover, SOCS2 was required for this inhibitory effect for both LXA_4_ and L-kynurenine (hatched bars – SOCS2-KO versus solid bars – WT DC’s). This set of results led us to suggest that LXA_4_ and L-kynurenine share a common inhibitory target and, possibly, an intracellular pathway that depends on the expression of SOCS2.

### L-kynurenine Induces SOCS2 Expression by Mouse Spleen-derived Dendritic Cells

**Figure 2:**
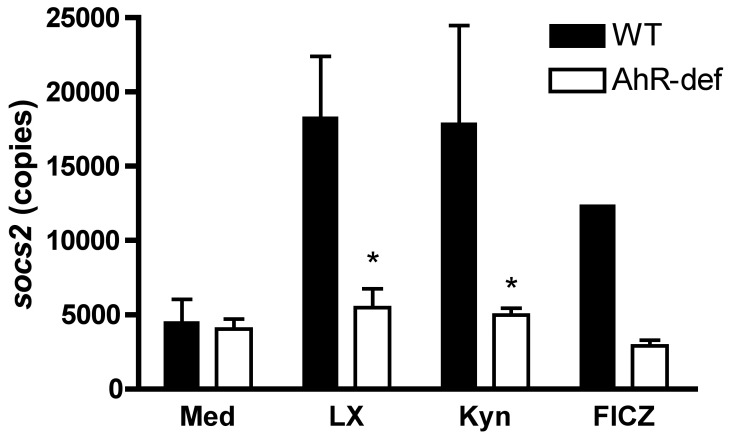
*socs2* mRNA expression in spleen-derived dendritic cells exposed to LXA_4_, L-kynurenine and FICZ is AhR-dependent. WT (filled bars) or AhR-deficient (empty bars) spleen-derived dendritic cells were isolated and incubated for 4 hrs in the presence of medium alone or with LXA_4_ (3 nM), L-kynurenine (60 µM) or FICZ (4 µM). Total RNA was then obtained, followed by real-time RT-PCR for *socs2*. Data shown represents expression values normalized against β-actin expression. Asterisks indicate statistically significant differences (*p*<0.05) when comparing WT *vs.* AhR group samples. Data shown is representative of four independent experiments performed.

We have previously shown that LXA_4_ triggered an AhR-dependent SOCS2 expression in mouse spleen-derived dendritic cells. Given the results shown above, that the inhibitory actions of L-kynurenine overlapped those seen when cells were exposed to LXA_4_ and that SOCS2-deficient cells failed to show L-kynurenine-mediated inhibition of pro-inflammatory cytokine production, we asked whether there is significant induction of *socs2* gene expression in dendritic cells exposed to L-kynurenine. As can be seen in Figure 2, L-kynurenine induced significant up-regulation of SOCS2 message levels. Importantly, SOCS2 induction by L-kynurenine was completely abolished in AhR-deficient cells. 6-formylindolo[3,2-b] carbazole (FICZ), a tryptophan metabolite used as a prototypical AhR ligand [21], also induced *socs2* gene expression in an AhR-dependent manner. Taken together, this set of results clearly indicates that AhR is a component of the pathway involved in SOCS2 induction by L-kynurenine in dendritic cells, in agreement with our previous experimental observations with LXA_4_.

### LXA_4_ and L-kynurenine Induce AhR Nuclear Translocation

**Figure 3:**
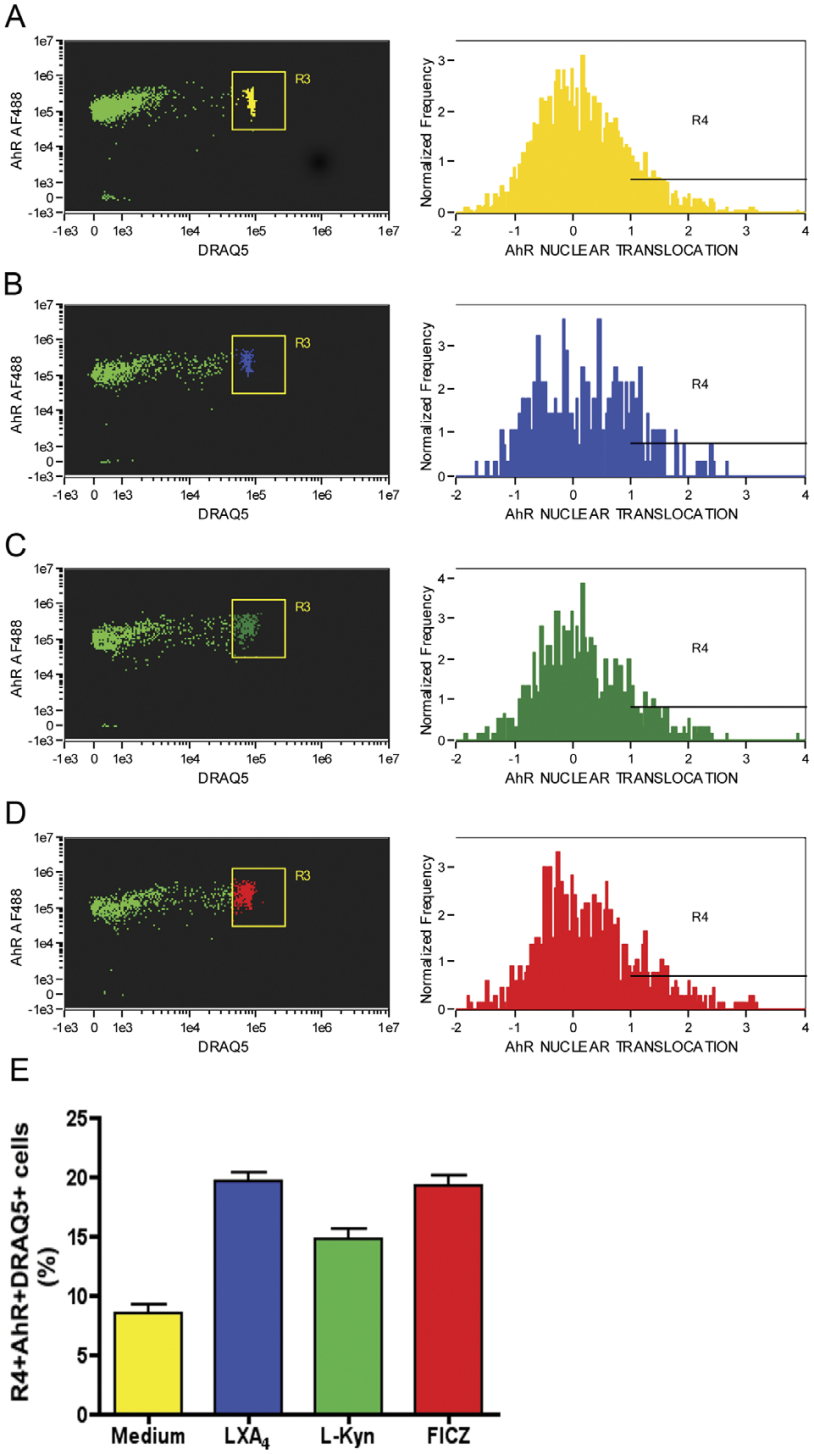
LXA_4_ and L-kynurenine mediate AhR nuclear translocation in spleen-derived CD11c+ cells. Collagenase-digested, low-density, spleen-derived cells were incubated in the presence of medium alone (yellow), or with FICZ (4 µM), LXA_4_ (3 nM) or L-kynurenine (60 µM) for 150 minutes followed by staining for CD11c, AhR and DNA (Draq5–nuclear counterstaining) and analyzed using ImageStream. Data shown indicates the CD11c+AhR+ cells gated for analysis of Draq5+AhR+ staining overlay histograms. Cells analyzed were incubated with medium alone (A), LXA4 (B), L-kynurenine (C) and FICZ (D). Panel E shows a summary of the data shown in panels A–D. Data is representative of three independent experiments performed.

**Figure 4:**
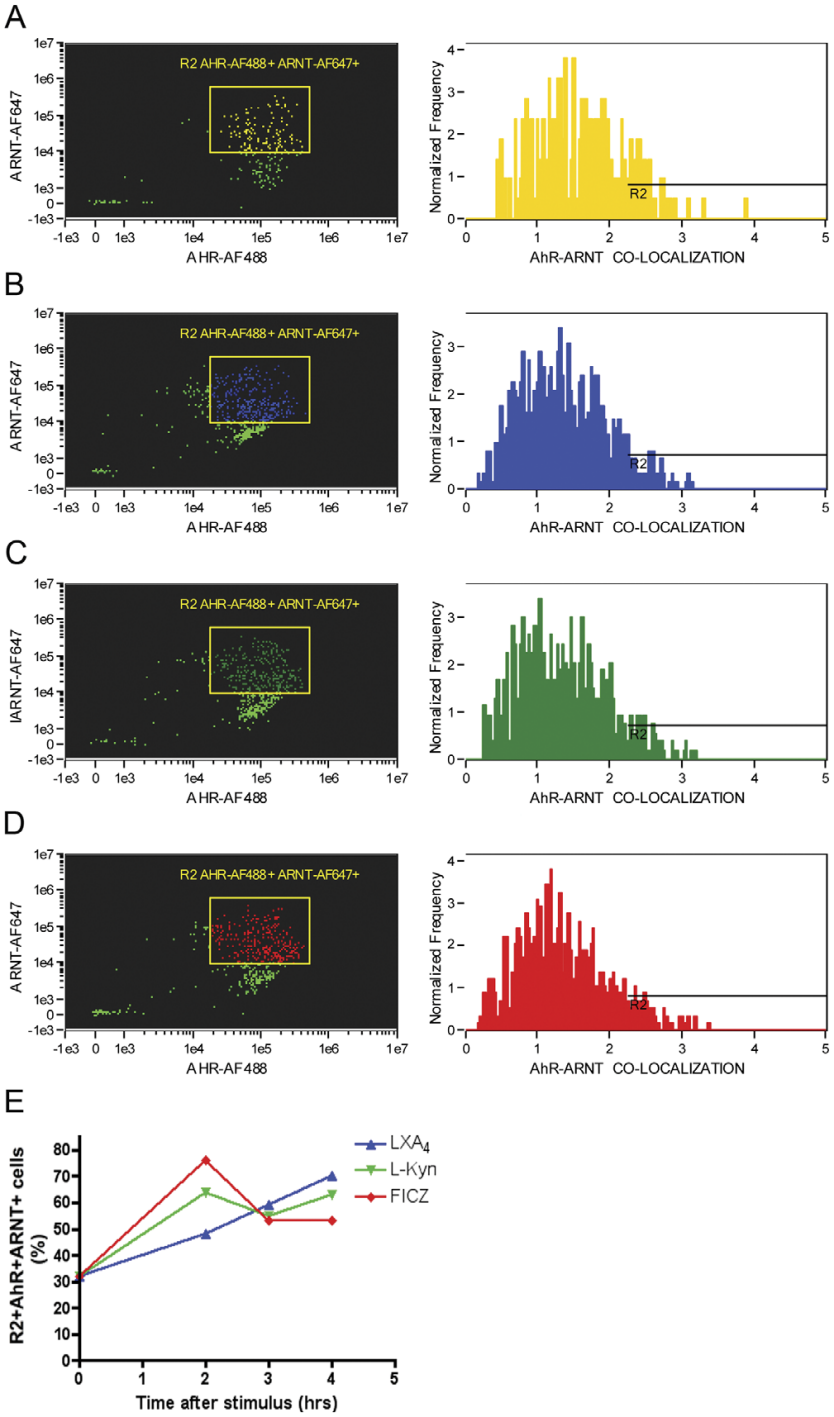
AhR and ARNT intracellular co-localization is induced after exposure to LXA_4_ and L-kynurenine. Collagenase-digested, low-density, spleen-derived cells were incubated in the presence of medium alone (yellow), or with FICZ (4 µM), LXA_4_ (3 nM) or L-kynurenine (60 µM) for 150 minutes followed by staining for AhR and ARNT and analyzed using ImageStream. Data shown indicates the AhR+ARNT+ cells gated for analysis of AhR+ARNT+ staining overlay histograms. Cells analyzed were incubated with medium alone (A), LXA_4_ (B), L-kynurenine (C) and FICZ (D). Panel E shows a summary of the data shown in panels A–D. Data is representative of three independent experiments performed.

Prototypical AhR ligands, such as dioxins, are thought to migrate through the cellular membrane and associate with AhR, which uncouples from chaperones, such as HSP90 [22]. Following, heterodimerization of AhR and ARNT takes place at the nuclear membrane, mediating its nuclear translocation [23]. Once in the nucleus, those heterodimers are thought to bind to specific promoter sequences, termed DRE [24]. In order to evaluate whether either LXA_4_ or L-kynurenine exposure initiates nuclear translocation of AhR, we performed ImageStream analysis of spleen-derived CD11c+ dendritic cells (Figure 3) after 2.5-hrs incubation in the presence of medium alone (Figure 3A), LXA_4_ (Figure 3B), L-kynurenine (Figure 3C) and the prototypical AhR-ligand, FICZ (Figure 3D). The results showed evident nuclear translocation in all groups (summarized in figure 3E), except for control cells incubated in medium alone. To further establish AhR as a molecular component of this shared anti-inflammatory pathway, we performed ImageStream analysis examining AhR/ARNT co-localization. Figure 4 indicates the frequency of AhR/ARNT co-localization among spleen-derived CD11c+ cells exposed to medium alone, LXA_4_, L-kynurenine and FICZ (Figure 4A–D). The results indicate a consistent up-regulation around 2 hrs after stimulation of AhR/ARNT heterodimers in cells treated with LXA_4_, L-kynurenine and FICZ, but not in control medium group (Summarized in figure 4E). In summary, the results shown here suggest that a common intracellular pathway is shared by both LXA4 and L-kynurenine; upon AhR ligand stimulation, nuclear receptor translocation occur leading to its subsequent dimerization with ARNT, in agreement with previous molecular interactions described for AhR [21]–[25].

### LXA_4_ and L-kynurenine Trigger DRE-dependent Gene Expression in Dendritic Cells

**Figure 5:**
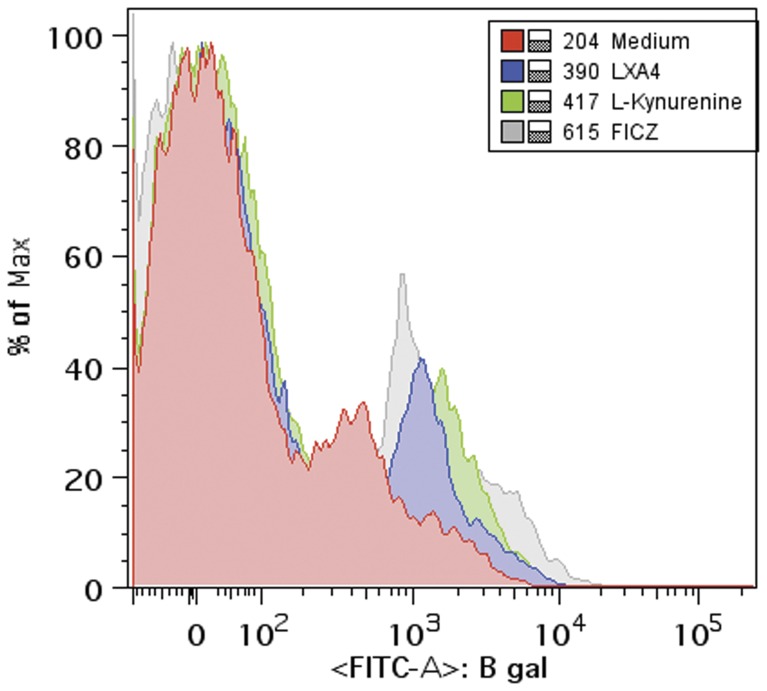
LXA_4_ and L-kynurenine exposure trigger DRE-mediated gene expression. LacZ transgenic mouse spleen-derived CD11c+ cells were purified and exposed to medium alone, or with LXA_4_ (3 nM), L-kynurenine (60 µM) or FICZ (4 µM) for 120 min, followed by staining with CD11c, CD8α and incubated with the beta-galactosidase substrate for 1 min at 37°C. Cells were then analyzed by flow cytometry. Data shown is representative of three independent experiments performed. Legend indicates the sample identification followed by its mean fluorescence intensity at the FL1-channel. Histograms were normalized and presented as % of maximum fluorescence, according to FlowJo analysis software.

The results shown so far delineate the events triggered after exposure to both LXA_4_ and L-kynurenine in mouse spleen-derived dendritic cells that ultimately lead to inhibition of cytokine production in response to pro-inflammatory stimuli. To do so, induction of SOCS2 via activation of AhR seems to be required. Moreover, both ligands can initiate AhR heterodimerization with ARNT and subsequent nuclear translocation. As seen with exogenous AhR ligands, such as dioxins, once the AhR/ARNT is localized in the nucleus, it binds to DRE sequences found within several gene promoter regions initiating a transcription program that includes several genes, including SOCS2. We therefore, pursued to examine whether LXA_4_ and L-kynurenine triggers AhR-dependent DRE-driven gene expression in vitro and in vivo. To do so, we took advantage of a transgenic mouse model in which beta-galactosidase is expressed under a promoter region containing three tandem DRE motifs [25]. This model animal model has been highly useful to identify the role of AhR in toxicological responses to exposure to environmental pollutants, such as dioxins. In order to determine which dendritic cell subsets up-regulate beta-galactosidase enzymatic activity, we used an adapted flow cytometry method, in which cleavage of the substrate by beta-galactosidase releases a fluorescent marker that can be detected by the cytometer. As shown in figure 5 (in vitro stimulated, purified spleen-derived dendritic cells), exposure to LXA_4_, L-kynurenine and FICZ induced up-regulation of DRE-driven beta-galactosidase enzymatic activity, which is evidenced by the increased detection of the fluorescent metabolite. LXA_4_ produced a steady induction of beta-galactosidase, as early as 1 hr after stimulation up to 12 hrs thereafter. On the other hand, both L-kynurenine and FICZ peaked beta-galactosidase activity at 2 and 4 hrs, declining afterwards (not shown). Suggesting that a potential limiting factor for AhR-dependent gene induction, i.e. AhR/ARNT dissociation, might have differentiated rates among several ligands.

### L-kynurenine and LXA_4_ Triggers SOCS2-dependent TRAF6 Poly-ubiquitinylation and Proteasomal Degradation in Mouse Spleen-derived Dendritic Cells

**Figure 6:**
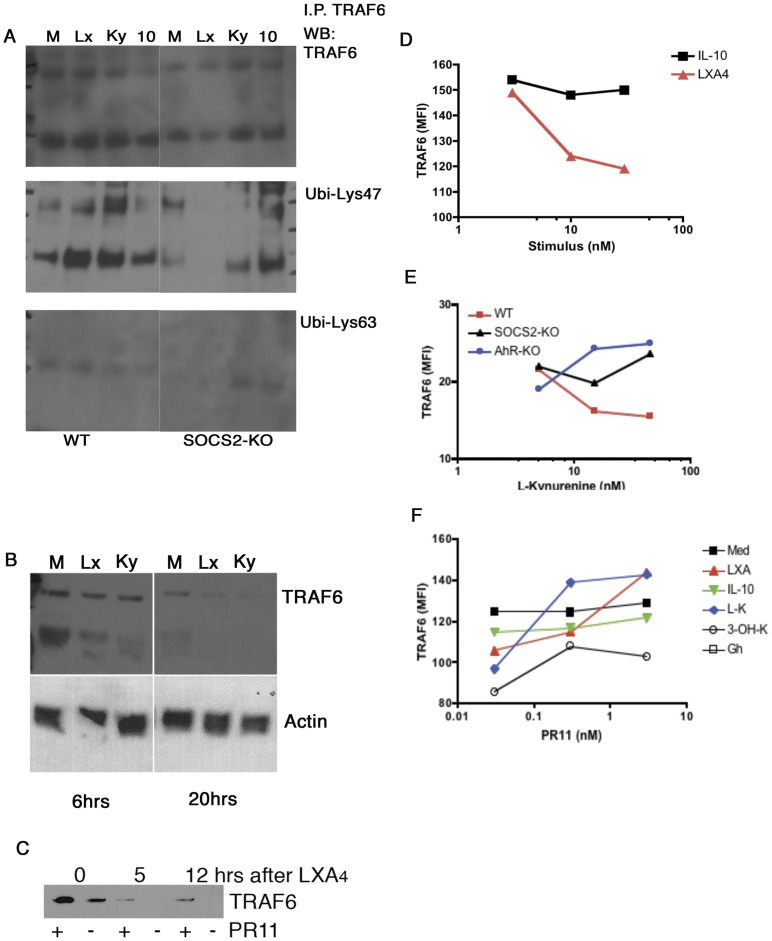
TRAF6 poly-ubiquitinylation and proteasomal degradation is triggered by DC exposure to LXA_4_ and L-kynurenine. WT spleen-derived DCs were cultured in the presence of medium alone (M) or with LXA_4_ (Lx) (1000 ng/mL), L-kynurenine (Ky) (60 µM) or IL-10 (10) (100 ng/mL) for 6 hrs. Cells were then homogenized and immuno-precipitated with an anti-TRAF6-agarose-bead mAb. The precipitates were analyzed by western blot for the presence of TRAF6, poly-ubiquitin (Lys47) and poly-ubiquitin (Lys63). (B) Total TRAF6/beta-actin western blot analysis in WT DC cell homogenates after incubation with medium alone (M), LXA_4_ (Lx) (1000 ng/mL) or L-kynurenine (Ky) (60 µM) for the indicated time points. (C) Time-course, western blot detection of total TRAF6 in spleen-derived cells incubated with LXA_4_ (3 nM) with or without pre-exposure to the proteasomal inhibitor PR11 (3 nM for 90 mins). (D) WT spleen-derived cells were incubated with increasing concentrations of IL-10 (black squares) or LXA_4_ (red triangles) for 6 hrs, followed by fixation/permeabilization and staining for CD11c and TRAF6 and analyzed by flow cytometry. Data shown represent plotted MFI’s for TRAF6 staining in CD11c+ cells. (E) WT (squares), SOCS2-KO (triangles) or AhR-deficient (circles) spleen-derived cells were purified and incubated with medium alone or with increasing concentrations of L-kynurenine (6–60 µM) for 6 hrs, cells were then fixed, permeabilized and stained for CD11c and TRAF6. The mean fluorescence intensity of TRAF6 staining within CD11c+ cells was then analyzed by flow cytometry. (F) WT spleen-derived cells were pre-incubated with PR11 (0.03 to 3 nM), followed by incubation with medium alone (filled squares) or with LXA_4_ (upright triangles –3 nM), IL-10 (inverted triangles –100 ng/mL), L-kynurenine (diamonds –60 µM), 3-OH-kynurenine (circles –3 µM) and growth hormone (empty squares –100 ng/mL) for 6 hrs. Cells were then fixed, permeabilized and stained for CD11c, CD8α and TRAF6. Cells were then analyzed by flow cytometry for their TRAF6 mean fluorescence intensity within the CD11c+CD8α+ subset. Data shown is representative of three independent experiments performed.

So far, our data support a common regulatory pathway initiated by exposure to LXA_4_ or L-kynurenine. The intracellular events identified here indicate that AhR is one of the intracellular signaling elements. Ligand exposure triggered its dimerization with ARNT, nuclear translocation and subsequent triggering of DRE promoter carrying genes (which includes *socs2*). Importantly, the anti-inflammatory actions of both ligands were shown to be dependent on SOCS2. Another critical overlap among the effects of both ligands is the broad suppressive effect over TRAF6-dependent responses (i.e. TLR, IL-1R). Therefore, we hypothesized that the AhR-triggered SOCS2 activity would be targeting TRAF6 to inhibit of signal transduction after pro-inflammatory receptor activation. To address this, we considered two main mechanisms by which SOCS proteins regulate signaling: allosteric blockage and proteasomal degradation of polyubiquitinylated targets. In order to test this hypothesis, we exposed spleen-derived dendritic cells to medium alone, LXA_4_, L-kynurenine or IL-10 (a immunomodulatory mediator that is SOCS2-independent) after several time points and tracked TRAF6 status by immune-precipitation/western blot as well as intracellular flow cytometry analysis (Figure 6). We observed that immune-precipitation of TRAF6 co-precipitated Lys47-linked poly-ubiquitin chains after exposure to LXA_4_ (Lx) and L-Kynurenine (Ky) but low levels in medium alone (M) or after exposure to IL-10 (10). Interestingly, no detection of Lys63-linked poly-ubiquitin chains was found under the conditions tested here (Figure 6A). Importantly, while WT DCs showed co-precipitation of TRAF6 with Lys47-linked poly-ubiquitin chains, SOCS2-deficient cells showed only moderate co-precipitation of Lys47-linked poly-ubiquitin chains with TRAF6 after IL-10, but not after LXA_4_ or L-kynurenine, suggesting that the cytokine may target TRAF6 through a SOCS2-independent pathway (Figure 6A). Figure 6B shows that detection of TRAF6 is significantly reduced at 20 hrs after LXA_4_ or L-kynurenine exposure in vitro. To further confirm that LXA_4_ and L-kynurenine trigger a proteasomal pathway that targets TRAF6, we pre-exposed spleen-derived dendritic cells with a proteasome inhibitor, the peptide PR11 (low-toxicity, reversible blocker of the ubiquitin recognition domains of the proteasome [26]). Figure 6C shows that the intracellular levels of TRAF6 are partially preserved when cells were exposed to PR11 prior to LXA_4_. To further confirm this set of findings through an independent technique (intracellular flow cytometry TRAF6 staining) we exposed WT DCs to LXA_4_ or IL-10 for 4 hrs and performed intracellular staining for TRAF6. The data shown in Figure 6D indicate a significant decline in the TRAF6 MFI for CD11c+ cells after LXA_4_, but not IL-10. Additionally, L-kynurenine drove an AhR/SOCS2-dependent decrease of TRAF6 intracellular levels in CD11c+ cells in a dose-dependent manner (Figure 6E). Furthermore, while control cells incubated in the presence of LXA_4_ or L-kynurenine alone showed significant decrease of TRAF6 MFI, pre-treatment with PR11rescued TRAF6 expression normal levels (Figure 6F). IL-10 or growth hormone (Gh) – a mediator that we have previously shown to induce SOCS2 expression in spleen-derived DCs [18], [27] – treatment did not affect the expression levels of TRAF6 detected. On the other hand, 3-OH-kynurenine (another tryptophan metabolite derived from IDO activity) also caused reduced detection of TRAF6, an effect that could be only partially restored by PR11 treatment (Figure 6F), however, in contrast to L-kynurenine this metabolite showed increased cytotoxicity.

Taken together, the results shown here provide compelling evidence that AhR/SOCS2-mediated proteasomal degradation of TRAF6 is a common pathway shared by both LXA_4_ and L-kynurenine.

### Lipoxin-triggered, SOCS2/AhR-dependent Proteasome Activity is Critical for Control of Inflammatory Responses during in vivo *Toxoplasma gondii* Infection

**Figure 7:**
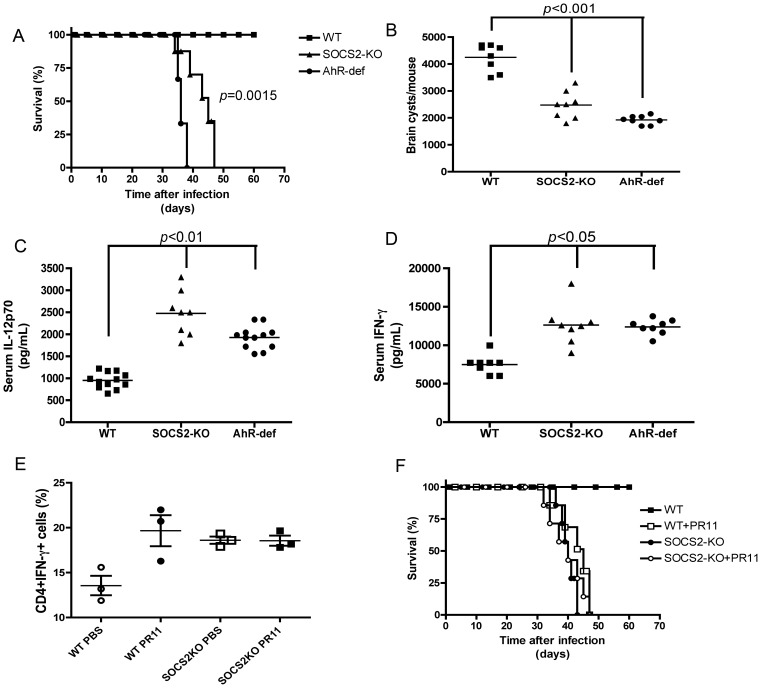
SOCS2 and AhR are required for modulation of host immune response during infection with *T. gondii*. WT (squares), SOCS2-KO (triangles) or AhR-deficient (circles) mice (*n* = 8 mice/group) were infected i.p. with 20 cysts of *T. gondii* (ME49 strain). Animals were monitored for (A) mortality, (B) cyst formation 30 days after infection, (C) serum levels of IL-12p70 at 5 days after infection and (D) serum levels of IFN-γ at day 7 after infection. (E–F) WT (circles) or SOCS2-KO (squares) mice were infected as described above, from 12 through 30 days after infection animals received daily i.p. injection of PBS (empty symbols) or 1 µg of PR11 (filled symbols). 30 days after infection, animals (*n = *3 mice/group) were sacrificed and antigen-specific CD4+T cell IFN-γ production analyzed by intracellular cytokine staining and flow cytometry. Cells were acquired and gated for CD3+ cells, followed by analysis of the frequencies of CD4 versus IFN-γ positive cells. The frequencies of IFN-γ+ among the total CD3+CD4+ cells are shown (E). Mortality was monitored throughout the acute and early chronic infection up to 60 days after inoculation (F). Data shown is representative of at least three independent experiments performed. *p* values are indicated for statistically significant differences between WT control groups versus SOCS2-KO or AhR-deficient strains.

Endogenously generated lipoxins control pro-inflammatory responses through SOCS2 during *T. gondii* infection [8]. In the absence of lipoxins or SOCS2, infection with *T. gondii* is lethal due to aberrant uncontrolled inflammation [8]. We hypothesized that TRAF6 proteasomal degradation is a downstream target for lipoxin-mediated control of pro-inflammatory responses during in vivo infection. We initially asked whether both AhR and SOCS2 would modulate counter-regulatory response during *T. gondii* infection. Figure 7A show that AhR-deficiency, similar to what we previously observed with SOCS2-KO mice, leads to mortality at around 45–50 days after infection with *T. gondii*. Brain cyst loads showed significantly lower counts in both SOCS2-KO and AhR-deficient mice. Moreover, serum levels of the protective pro-inflammatory cytokines, IL-12p70 (Figure 7C) and IFN-γ (Figure 7D) were significantly higher in both of the genetically mutant mouse lineages after infection. Taken together, these results establish that during in vivo *T. gondii* infection, lipoxin-mediated activation of AhR and induction of SOCS2 expression leads to a counter-regulatory pathway that inhibits excessive type 1 pro-inflammatory cytokine production in vivo.

To further establish the role of lipoxin-induced proteasomal degradation in controlling immune responses to in vivo infection, we infected WT mice with *T. gondii*, with or without daily treatment with PR11, starting from day 11 after infection – a time-point previously found to be the time at which increases in serum levels of LXA_4_ are detectable [8]. At this time point, most T cell priming is thought to have occurred. Moreover, direct testing of the effect of proteasome inhibition on CD8+ T cell during LCMV infection showed that such treatment did not affect expansion of CD8+T cells specific for LCMV-derived peptides (D. Hildeman, unpublished data). Figures 7E–F shows that proteasome inhibition during *T. gondii* infection mirrored the uncontrolled pro-inflammatory phenotype seen in both 5-lipoxygenase (LO)-deficient and SOCS2-deficient mouse strains [8], [18], including: (Fig. 7E) high levels of circulating CD4+CD3+IFN-γ+ T cells and (Fig. 7F) mortality occurring around 40–50 days after inoculation. Taken together, these data clearly establish TRAF6 as critical downstream target for proteasomal degradation mediated by endogenous lipoxin-stimulated SOCS2. Thus, lipoxin-mediated degradation of these key pro-inflammatory signaling adaptor proteins is essential for tight control of the innate immune response to infection with *T. gondii*.

**Figure 8:**
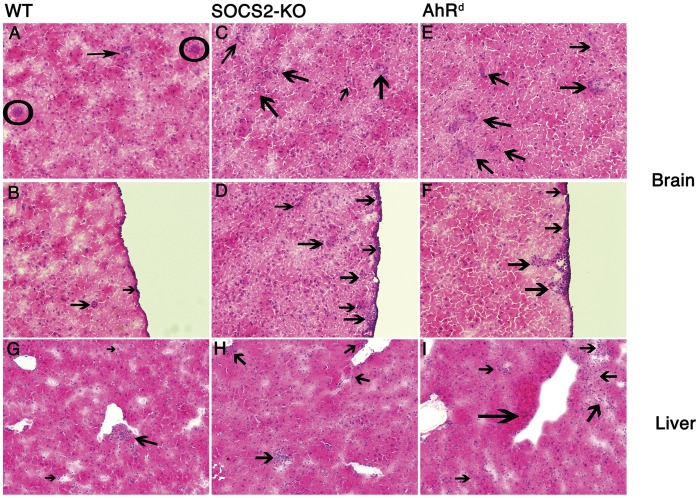
Increased brain inflammation during chronic *T. gondii* infection in the absence of SOCS2 and AhR in mice. WT (A, B and G), SOCS2-KO (C, D and H) and AhR*^d^* (E, F and I) mice were infected as described above. At 30 days after infection, animals were sacrificed, brains (A–F) and livers (G–I) removed and processed for H&E staining. Circles indicated parasite cysts in brain sections (Panel A). Arrows indicate inflammatory cell infiltrates. Images are representative of each group (*n = *3 mice per group). Magnification 10×.

To further substantiate the biological relevance of LXA_4_/SOCS2/AhR pathway in controlling pro-inflammatory response during infection with *T. gondii*, we perform histopathological analysis of the brain and liver tissues obtained from WT, SOCS2-KO and AhR*^d^* mice at 30 days after infection. Figures 8A–B shows typical inflammation in chronically infected WT mice. Panel 8A displays two parasite cysts (circles) and inflammatory infiltrate (indicated by an arrow) which are focal and perivascular with no significant meningitis (Figure 8B). On the other hand, brain sections from both SOCS2-KO (Fig. 8C–D) as well as AhR*^d^* (Fig. 8E–F) show significantly increased and diffuse inflammatory cell infiltrates, including atypical meningitis (Figs. 8D and F, respectively). In addition, the liver sections from infected WT, SOCS2-KO and AhR*^d^* mice showed a similar trend with regard to inflammatory cell infiltration: perivascular/focal mononuclear infiltrates (Figs. 8G to I). In summary, this set of results provides further support for the pivotal role of the LXA_4_/SOCS2/AhR pathway in preventing damage to host tissues during the chronic phase of *T. gondii* infection.

## Discussion

Counter-regulation of immune responses mediated by both lipoxins and IDO-dependent mechanisms have been found to be relevant in a growing list of infectious, chronic inflammatory and auto-immune diseases, including toxoplasmosis, HIV infection, leishmaniasis, tuberculosis, cystic fibrosis, asthma and periodontitis [14], [27]. Importantly, the intracellular mechanisms by which such relevant anti-inflammatory actions occur are poorly understood. While several potential pathways have been suggested to explain IDO-dependent actions [28], the intracellular mechanism of lipoxins is still elusive. Given the common target aimed by both ligands, we hypothesize here that a shared regulatory pathway must take place. In fact, we show here several lines of evidence that clearly demonstrate that both LXA_4_ and L-kynurenine trigger AhR-driven SOCS2 expression, including AhR-dependent induction of SOCS2, increased co-localization of AhR with ARNT, nuclear translocation of AhR and activation of gene expression in a DRE-induced manner. In turn, AhR-triggered SOCS2-mediated Lys47-linked poly-ubiquitin chain ligation of TRAF6, a well-known marker for proteasome-targeted degradation. Conversely, pre-exposure to inhibitors of proteasome recognition of such poly-ubiquitin chains, led to abolishment of TRAF6 degradation.

Although our data provide clear evidence of the downstream events initiated after LXA_4_ and L-kynurenine exposure, the biochemical nature of the recognition and binding to AhR is not established yet. It has been demonstrated that AhR is found in the cytosol associated with HSP90 in a non-active state [22]. In the presence of dioxins, AhR is released from HSP90 and form a stable heterodimer with the nuclear transporter ARNT. Studies using tryptophan and its metabolites have shown their binding to AhR, including FICZ, another commonly used prototypical exogenous ligand for AhR [29]. Although there is no direct evidence of L-kynurenine binding to AhR, our results indicated a strong cause-effect correlation between the two. A parallel case can be traced between LXA_4_ and AhR, where all evidence point to a strict cause-effect relationship, no direct binding data has been shown. One possible explanation is the volatile nature of LXA_4_ and the relative low-sensitivity of the binding assays available. Another possibility is that in both the cases of LXA_4_ as well as L-kynurenine, a new molecule is generated or a significantly modified version of the ligands reach AhR. In any case, those unanswered questions remain to be further explored in future studies.

Another important aspect of this pathway is the putative formation of an ubiquitin ligase complex by SOCS2 targeting Lys47-linked poly-ubiquitin chain ligation onto TRAF6 for proteasome degradation. TRAF6 can be ubiquitinylated with mono- or Lys63-linked poly-ubiquitin chains, typical markers of activation pathways, in which ubiquitinylated TRAF6 leads to binding to other signaling components, such as TAK6 among others [30]. The molecular basis that determine one form (proteasome directed) versus the other (activation associated) are not clear at the moment. It is possible that a second component is mediating the ubiquitin ligation changes or that TRAF6 becomes susceptible to Lys47-linked poly-ubiquitinylation after some form of post-translational modification, such as tyrosine phosphorylation – a key element for target protein recognition for SOCS proteins. Although there are no reports on TRAF6 phosphorylation or a potential biological function associated with TRAF6 phosphorylation, it is a possible scenario that has not been completely ruled out yet. Nevertheless, the cause-effect relationship between elevated expression of SOCS2 and proteasome degradation of TRAF6 is undeniable. Furthermore, all those effects are strongly involved in the mediating the anti-inflammatory actions mediated by both LXA_4_ and L-kynurenine.

A fundamental question in host-pathogen interactions is whether the immune system is properly activated to eradicate pathogens and if this response is appropriately controlled to prevent damage to the host during control or elimination of infection [27]. Microbial recognition is a critical first step that is central to both innate and adaptive immunity. DCs signal the presence of microbes via pattern recognition receptors such as the Toll-like receptors (TLR) [31]–[34] and respond by secreting cytokines and other effector molecules, which are critical for immune activation and dictating the type of immune response. During infection with *T. gondii* (and infections with other intracellular pathogens) the production of IL-12, IFN-γ and TNF by DCs is essential to control pathogen growth [27], [35], [36]. IL-12 and TNF are potentially toxic when produced in excess; indeed, their production is tightly regulated by a remarkably large number of different mechanisms [1], [27], [32]. In toxoplasmosis, the pro-inflammatory process is counterbalanced by the simultaneous induction of critical, non-redundant counter-regulatory mediators, including IL-10 and LXs [18], [27]. Mice with a genetic deficiency in LX production succumb to lethal inflammatory responses during toxoplasmosis [8]. In turn, the biological importance and non-redundant nature of the proteasome pathway to the mechanism of action of LXs is underscored by the finding that proteasome inhibition during toxoplasmosis prevents the regulatory actions of LX, restoring TLR and cytokine receptor signaling in vivo and causing uncontrolled pro-inflammatory cytokine production, aberrant leukocyte infiltration and elevated mortality. Interestingly, we found that during mouse toxoplasmosis, IDO did not exert an evident immuno-regulatory function, as seen during other infectious diseases. Instead, IDO perform a critical microbiostactic function, possibly due to tryptophan depletion, allowing macrophages to prevent intracellular growth of *T. gondii* [14]. Therefore, it is not feasible to examine the immune-modulation caused by released L-kynurenine during *T. gondii* infection.

It has become clear in recent years that uncontrolled inflammatory responses are central to the pathogenesis of a wide range of diseases causing an enormous burden of morbidity and mortality in the world at large. The results presented here suggest that degradation of TRAF6 is a shared/redundant anti-inflammatory pathway that provides a promising biochemical target for drug development aimed at modulating inflammatory processes in these diseases.

## Materials and Methods

### Mice

Wild-type controls (C57BL/6) and AhR-deficient (AhR*^d^*) mice were obtained from The Jackson Laboratory. SOCS2-deficient mice were kindly provided by Drs. W.S. Alexander (The Walter and Eliza Hall Institute for Medical Research, Parkville, Victoria, Australia). All animals were bred and maintained under pathogen-free conditions at a Children’s Hospital Medical Center animal facility (Cincinnati, OH) under a protocol approved by the Children’s Hospital Medical Center Animal Care and Use Committee. All experiments shown here were previously approved under the above protocol.

### Infections

*T. gondii* cysts (ME49 strain) were recovered from brain homogenates from chronically infected mice and suspended in PBS. Animals were experimentally infected by intraperitoneal injection with 20 cysts per mouse in vehicle (0.2 ml/animal). For in vivo proteasome inhibition, animals received 0.1 µg of PR11 (BIOMOL) i.p. at days 13, 15, 17, 19 and 21 after infection.

### Cytokine Determination

IL-12 p70, TNF-α and IFN-γ levels were measured using commercial ELISA kits (BD biosciences). For *in vitro* experiments, splenic DCs were partially purified as described [32]. Briefly, spleens harvested from mice were digested with Liberase CI (Roche Biochemicals, Indiana, IN). Low-density (LOD) leukocytes were then obtained after centrifugation in a dense-BSA gradient and CD11c^+^ cells were further purified using magnetic bead-conjugated antibody (Miltenyi Biotech, Auburn, CA). A representative dot-plot of the cells after purification is shown in figure S1. After washing in PBS, cells were resuspended in RPMI 10% FCS at 1×10^6^/ml in 96-well plates (Corning, Corning, NY) and test stimuli were added as indicated for each experiment. For *in vivo* experiments, mice (*n* = 5–10) were infected intraperitoneally (i.p.) with 0.2 ml of PBS and 5 and 7 days after infection were bled for assessment of cytokine levels.

### ImageStream Analysis

Spleen-derived CD11c+ dendritic cells (obtained as described above) were exposed to the following stimulations; LXA_4_ (3 nM), L-kynurenine (60 µM) and FICZ (100 nM). 5×10^6^ cells were placed in 6 well flat-bottom plates for 2.5 hrs *in vitro* incubation in RPMI 10% FCS. The cells were then washed and prepared for staining. AhR and ARNT (Santa Cruz) primary antibodies were used to stain cells after stimulation. AlexaFluor 488- and AlexaFluor 647-conjugated anti-rabbit or rat IgG pAbs were used as secondary antibodies. Draq5 (Cell Signaling) was used as nuclear dye. Amnis ImageStream system was used to study AhR cellular localization and AhR/ARNT molecular interactions.

### Flow Cytometry-based Beta-galactosidase Assay

Spleen-derived dendritic cells were purified after collagenase digestion followed by magnetic bead-conjugated anti-CD11c purification. Cells were cultured for 1–12 hrs in the presence of medium alone or FICZ, LXA_4_, or L-kynurenine (100 ng/ml). Cells were re-suspended and stained for CD11c, CD8α and incubated for 1 min at 37°C in the presence of fluorescein di-V-galactoside (FDG). Cells were acquired in a LSRII cytometer and analyzed using FlowJo software.

### Cell Extracts and Immunoblot Assay

Whole cell extracts were generated by lysing DCs in buffer containing 50 mM HEPES pH 7.9, 0.25 M NaCl, 5 mM EDTA, 0.1% NP-40, 1 mM PMSF, 1× HALT™ protease inhibitor cocktail Pierce Biotechnology Inc. (Rockford, IL). For detection of phosphorylated-proteins, Na_3_VO_4_ and NaF, were added to lysis buffers. The immunoprecipitation was performed with TRAF6 antibody purchased from Santa Cruz Biotechnology Inc. (Santa Cruz, CA). The detection of protein expression by western blot was performed with primary antibodies against TRAF6 purchased from Santa Cruz Biotechnology Inc. (Santa Cruz, CA), poly-ubiquitin (Lys47) and poly-ubiquitin (Lys 63) mAb’s were purchased from Millipore.

### Real Time RT-PCR

Total RNA was isolated from murine DC culture using the Trizol LS reagent according to the instructions of the manufacturer. cDNA was synthesized with TaqMan Reverse Transcriptase (Applied Biosystems, Foster City, CA) and mRNA expression of cytokines (IL-12p35, IL-12p40, IL-23p19, IL-6, IFN-α3 and TNF) – and β-actin was analyzed by RT-PCR. Real-time RT-PCR was performed on an ABI-Prism 7000 PCR cycler (Applied Biosystems).

### Statistical Analysis

The statistical significance of differences in mean values between experimental versus control or vehicle treated samples was evaluated by means of Student’s t test. Differences were considered to be significant at *p*<0.05.

## Supporting Information

Figure S1 Flow cytometry phenotype of spleen derived dendritic cells. Spleens were digested with Collagenase D, followed by low-density gradient. The resulting cell suspensions were labeled with MACS beads-conjugated anti-CD11c mAb and purified using MACS columns. Cells were subsequently stained with PacificBlue-CD11c mAb and analyzed by flow cytometry. The dot plot shows a typical frequency of total CD11c+ cells obtained using this protocol.
(TIF)
