# Supplementary figures and images for: SOCS2-Induced Proteasome-Dependent TRAF6 Degradation: A Common Anti-Inflammatory Pathway for Control of Innate Immune Responses
Source: PLoS One. 2012 Jun 5;7(6):e38384. doi: 10.1371/journal.pone.0038384 (PMC3367914; doi:10.1371/journal.pone.0038384)

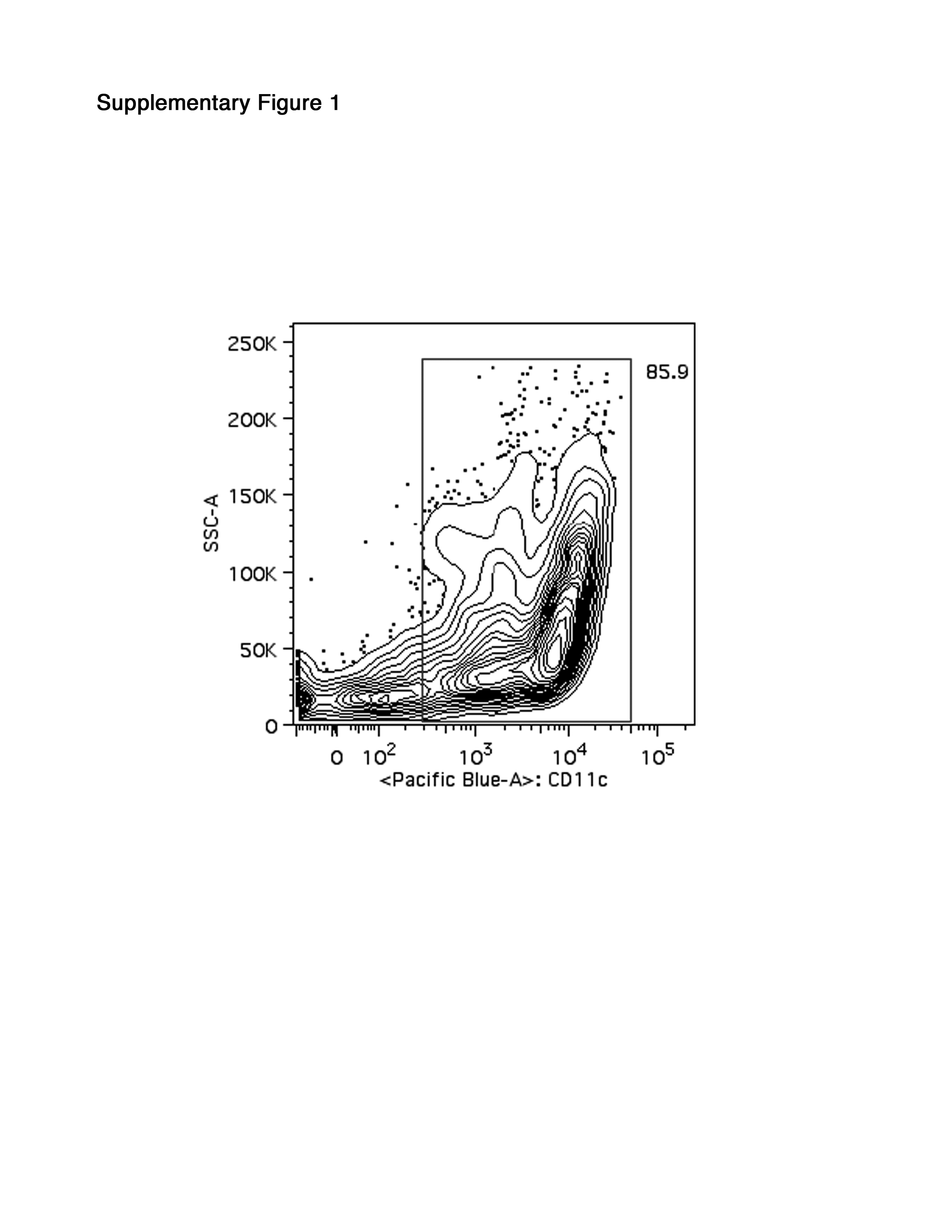

Supplement: Figure S1 — Flow cytometry phenotype of spleen derived dendritic cells. Spleens were digested with Collagenase D, followed by low-density gradient. The resulting cell suspensions were labeled with MACS beads-conjugated anti-CD11c mAb and purified using MACS columns. Cells were subsequently stained with PacificBlue-CD11c mAb and analyzed by flow cytometry. The dot plot shows a typical frequency of total CD11c+ cells obtained using this protocol. (TIF) [file pone.0038384.s001.tif]
